# Spatial Distribution of Brain PET Tracers by MALDI Imaging

**DOI:** 10.1021/jasms.4c00307

**Published:** 2025-03-12

**Authors:** Isabeau Vermeulen, Michiel Vandenbosch, Delphine Viot, Joel Mercier, Diego Asensio-Wandosell Cabañas, Pilar Martinez-Martinez, Patrick Barton, Ron M.A. Heeren, Berta Cillero-Pastor

**Affiliations:** $ The Maastricht MultiModal Molecular Imaging Institute (M4i), Division of Imaging Mass Spectrometry, Maastricht University, Universiteitssingel 50, 6229 ER Maastricht, The Netherlands; & Translational Science, DMPK, UCB Biopharma SRL, Chemin du Foriest, B1420 Braine-l’Alleud, Belgium; § Discovery Chemistry BE, UCB Biopharma SRL, Chemin du Foriest, B1420 Braine-l’Alleud, Belgium; % Department of Psychiatry and Neuropsychology, Maastricht University, Universiteitssingel 40, 6229 ER Maastricht, The Netherlands; ≫ Translational Science, DMPK, UCB Celltech, Branch of UCB Pharma S.A., 208 Bath Road, Slough, Berkshire SL1 3WE, United Kingdom; + Cell Biology-Inspired Tissue Engineering (cBITE), MERLN, Maastricht University, Universiteitssingel 40, 6229 ER Maastricht, The Netherlands

## Abstract

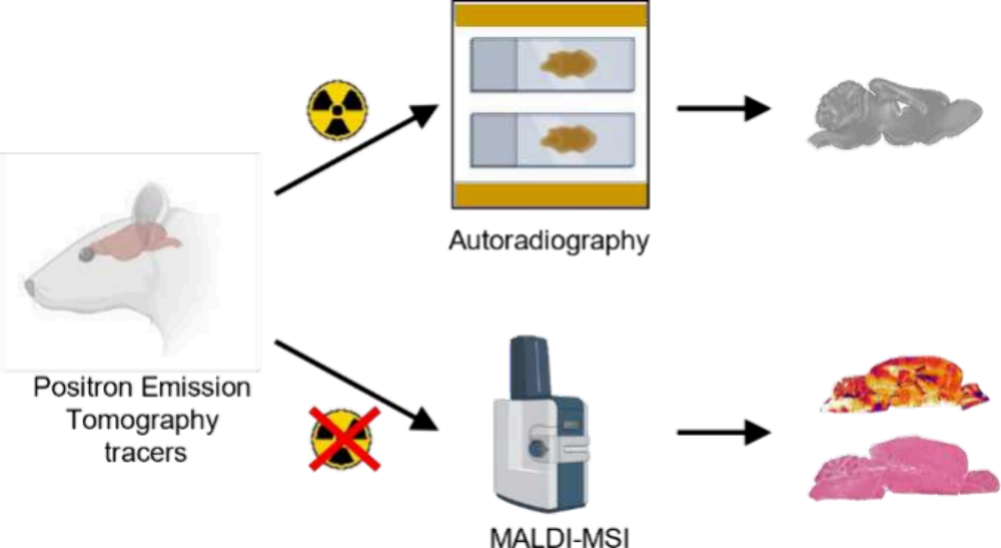

Evaluating tissue distribution of Positron Emission Tomography (PET) tracers during their development conventionally involves autoradiography techniques, where radioactive compounds are used for *ex vivo* visualization and quantification in tissues during preclinical development stages. Mass Spectrometry Imaging (MSI) offers a potential alternative, providing spatial information without the need for radioactivity with a similar spatial resolution. This study aimed to optimize a MSI sample preparation protocol for assessing PET tracer candidates *ex vivo* with a focus on two compounds: UCB-J and UCB2400. We tested different matrices and introduced washing steps to improve PET tracer detection. Tissue homogenates were prepared to construct calibration curves for quantification. The incorporation of a washing step into the MSI sample preparation protocol enhanced the signal of both PET tracers. Our findings highlight MSI’s potential as a cost-effective and efficient method for the evaluation of PET tracer distribution. The optimized approach offered here can provide a protocol that enhances the signal and minimizes ion suppression effect, which can be valuable for future evaluation of PET tracers in MSI studies.

## Introduction

Positron Emission Tomography (PET) is a noninvasive imaging technique broadly employed in clinical settings to detect and quantify PET radiotracers that binds to specific biological targets in a living body, such as enzymes and receptors. This diagnostic modality plays a role in disease detection within organs. Beyond its diagnostic capabilities, PET imaging also aids researchers to investigate whether newly developed molecules or medicines effectively reach their intended targets. Furthermore, it can offer direct insights into target occupancy.^1^ However, the process of identifying suitable PET tracer candidates presents several challenges due to factors such as the short *in vivo* half-life, cost, and feasibility of PET tracer synthesis.^2−6^

ADMET studies (absorption, distribution, metabolism, elimination, and toxicology) form a key element in PET tracer discovery and development, as they increase confidence in the prediction of human pharmacokinetic properties as well as the safety of tracer candidates. In particular, ADME studies are important as they provide more information about the tissue distribution of the novel PET tracers.^7^ Traditionally, information about the distribution of PET tracers is generally derived from two main approaches: either radioactive ’hot tracer’ or nonlabeled ’cold tracer’ LC-MS (liquid chromatography–mass spectrometry) analyses of organ or tissue homogenates or quantitative (whole-body) autoradiography (QWBA).^8,9^ In “hot tracer” LC-MS analysis, radioactive PET tracers are administered to rodents, followed by dissection and homogenization of tissues. The radioactive signal in different organs is then measured together with mass information using LC-MS to determine the quantity of the tracer and its metabolites present in specific organs.^9^ QWBA involves administration of a PET tracer labeled with ^3^H, ^14^C, or ^125^I to rodents. Whole-body sections are then exposed to phosphor plating to capture emitted photons, enabling imaging and quantification of tracer distribution throughout the body.^10^ Although these methods are informative, they have several limitations such as the need for radioactive labeling, which causes safety and logistical challenges. QWBA is also unable to differentiate between the parent molecule and its metabolites because it detects radioactivity without distinguishing the specific chemical identity of the compounds. Further, loss of spatial distribution in LC-MS, and the iterative process of evaluating several [^3^H]-labeled compounds in “hot tracer” LC-MS and QWBA before discovering a promising candidate limits these techniques.^11,12^

To address these limitations and open the possibilities for a safer and more efficient PET tracer discovery process, together with a decrease in costs, we investigated the possibility of using Mass Spectrometry Imaging (MSI). In particular, matrix-assisted laser desorption/ionization-MSI (MALDI-MSI) could be a promising and safe approach for studying the distribution of PET tracers because it does not require any (radioactive) labeling and provides high spatial resolution. Previous studies by Goodwin et al. used a solvent-free manual matrix application to allow MALDI-MSI detection of two PET tracers targeted at dopamine receptors 1 and 2 in the brain.^13^ Another study by Jacobsen et al. used Desorption Electrospray Ionization (DESI)-MSI to detect Cimbi-36 in the brain at spatial resolution of 225 μm.^14^

Building on previous research demonstrating the use of MSI for PET tracers, in this study, we demonstrate the effectiveness of using MALDI-MSI with higher spatial resolution (50 μm) for detecting and visualizing two distinct PET tracers in the brain: UCB-J and UCB2400 (Figure S1).^15,16^ UCB-J is a PET tracer that targets the synaptic vesicle glycoprotein 2A (SV2A), with reduced levels observed in various neurological disorders, including epilepsy. In contrast, UCB2400 is designed to bind to the dopamine 1 receptor, which plays a critical role in diseases such as Alzheimer’s and Parkinson’s. These two tracers were selected because they originate from distinct chemical families, characterized by different molecular structures, which may influence their behavior in MALDI-MSI experiments by affecting factors such as ionization efficiency, fragmentation patterns, and overall detection sensitivity. UCB-J features a pyridine and a fluorophenyl moiety, with a phenyl ring substituted by three fluorine atoms and a carbonyl group in a cyclic structure. UCB2400, on the other hand, includes a quinoline structure with a fused ring system, containing amine and carbonyl functional groups that can participate in hydrogen bonding. The presence of a methyl substituent further alters its physicochemical properties. These structural differences impact their behavior in biological systems, including their binding profiles and ability to cross the blood-brain barrier. Additionally, the logD (octanol–water) for UCB-J is 2.5, while UCB-2400 has a logD of 2.1. Although the difference in logD is not very high, it further reflects the variability in their physicochemical properties.

To achieve reliable and specific detection of these tracers, several parameters must be considered, including the choice of matrix, MS instrument, and the presence of other molecules in the brain tissue that may suppress ionization.

Ion suppression, a complex phenomenon involving various factors, can affect the limit of detection. One method to overcome this is to employ washing steps after cryosectioning. These steps involve the use of solvents to remove ion-suppressing molecules such as lipids and salts, which can interfere with the signal of interest. Previous studies have explored the use of various solvents such as ammonium acetate, chloroform, and ammonium formate for tissue washing before MALDI-MSI.^17−21^ However, for small molecules such as PET tracers, the choice of the washing solvent is critical. It is important that the solvent does not lead to compound delocalization.^19^ Therefore, selecting an appropriate washing solvent is crucial for ensuring the accurate and localized detection of PET tracers.

In this study, we developed and improved a MALDI-MSI workflow for the detection of PET tracers. The potential of MALDI-MSI for imaging PET tracers is substantial, as it does not require the extensive research typically involved in developing radioactively labeled compounds. This approach allows for rapid exploration of tracer distribution in tissues. The expectation in doing so is that it will substantially decrease the development burden in terms of cost, timelines, and attrition rate. While this study primarily focuses on the distribution aspect, the washing step and subsequent MALDI-MSI analysis could also be adapted to study metabolism. For example, MALDI-MSI could be employed to detect and localize metabolic products formed during tracer interactions with biological targets, offering valuable insights into metabolic pathways. Furthermore, MALDI-MSI can be extended in future studies to investigate the elimination of PET tracers and their metabolites, providing spatially resolved information on tissue clearance.

## Experimental Section

### Reagents and Solvents

2,5-Dihydroxybenzoic acid (DHB) and α-cyano-4-hydroxycinnamic acid ≥98% (α-CHCA) were purchased from Sigma-Aldrich. (Zwijndrecht, The Netherlands). Trans-2-[3-(4-*tert*-Butylphenyl)-2-methyl-2-propenylidene]malononitrile (DCTB) was purchased from Bioconnect (Huissen, The Netherlands). Ethanol, ultrapure water (LC-MS grade), methanol, acetone, dichloromethane (DCM), ethyl acetate (EtOAc), and chloroform were purchased from Sigma-Aldrich and used without further purification (Zwijndrecht, The Netherlands). The UCB-J and UCB2400 standards were provided by UCB Pharma (Brussels, Belgium). Gill’s hematoxylin and Entellan were obtained from Merck KGaA (Darmstadt, Germany). Eosine-Y, alcoholic was purchased from Avantor Performance Materials, B.V. (Deventer, The Netherlands). Coverslips were obtained from Thermo Scientific (Waltham, Massachusetts, U.S.A).

### Tissue Collection

All studies were carried out in accordance with internal (UCB Pharma) Ethical Committee and Animal Care Unit guidelines, the European Committee Criteria (Decree 2010/63/CEE) and the Animal Welfare Act (7 USC 2131). All experiments were performed on males Crl:CD-SD rats (Charles River). For control animals, rats were perfused with 60 mL 0.9% NaCl with 25 IU/mL heparin, prior to brain collection. The brain tissue was snap-frozen in a metal box on dry ice, placed in a Styrofoam box, and stored at −80 °C until cryosectioning. Control tissues were sectioned at a thickness of 12 μm at −21 °C in a Leica cryostat (Leica Microsystems, Wetzlar, Germany). The sections were thaw-mounted onto indium thin oxide (ITO)-coated glass slides (4–8 Ω resistance, Delta Technologies, U.S.A.) and stored at −80 °C. For homogenates preparation, six rats were perfused with 60 mL 0.9% NaCl with 25 IU/mL heparin. The brains (including the cerebellum) were then resected. Brain homogenization was performed using an Ultra-Turrax T25 with S25N tools at a speed of 24.000 t/min. Three brains were employed per UCB compound. For each homogenate, 100 mg per sample were used. Then, the UCB compound was added to each homogenate to obtain a volume of 1% of the total weight. Final concentrations were ranging from 1.6 μg UCB compound/g brain to 25 μg UCB compound/g brain. After spiking the homogenate with the compound, it was incubated for 30 min in a Thermoshaker (37 °C, 900 rpm) to allow compound penetration and binding. This incubation step was specifically designed to better mimic *in vivo* conditions for our limit of detection (LOD) experiment. Homogenates were pipetted into a gelatin mold (20% gelatin) and frozen at −80 °C as described previously by Lamont et al.^22^ Samples were kept overnight at −80 °C until cryosectioning. The homogenates were then sectioned at a thickness of 12 μm at −21 °C in a Leica cryostat (Leica Microsystems, Wetzlar, Germany) and thaw-mounted onto ITO slides. For dosed animals, six rats were intravenous dosed with the PET tracer UCB-J (3 mg/kg/2 mL/kg) in a vehicle 30% dimethylacetamide (DMA) in 20% kleptose HPB (hydroxypropyl beta-cyclodextrin) with an average animal weight of 302g ± 5. At 5- or 20 min postdose, animals were euthanized by carbon dioxide asphyxiation. Perfusion was done with 60 mL 0.9% NaCl with 25 IU/mL heparin prior to brain collection. The brain tissues were snap-frozen in a metal box on dry ice, placed in a Styrofoam box, and stored at −80 °C until cryosectioning. All tissues were sectioned at a thickness of 12 μm thickness and −21 °C using a Leica cryostat (Leica Microsystems, Wetzlar, Germany). The sections were thaw-mounted onto indium thin oxide (ITO)-coated glass slides (4–8 Ω resistance, Delta Technologies, U.S.A.) and stored at −80 °C until further analysis.

### MALDI Sample Preparation

#### On-Tissue Limit of Detection (LOD)

A 100 mM stock solution was prepared, dissolved in DMSO. Three different concentrations (50 μM, 10 μM, and 1 μM) of UCB-J and UCB2400 were prepared by diluting the stock solution in H_2_O and applied as 1 μL spots onto naïve sagittal rat brain sections. After the spots were dried in the desiccator (1 h until fully dried as the use of water-based solutions required a longer drying time), the sections were sublimed with a matrix of choice, CHCA, DHB, or DCTB. Table 1 provides an overview of the parameters used for the matrix application by sublimation.

**Table 1:** Sublimation methods

	CHCA	DHB	DCTB
amount	50 mg	50 mg	30 mg
solvent	2 mL acetonitrile/water (70:30)	2 mL acetone	2 mL methanol
temperature	180 °C	160 °C	160 °C
time	300 s	160 s	240 s

#### Washing Steps

A 100 mM stock solution was prepared, dissolved in DMSO. Three different concentrations (50 μM, 10 μM, and 1 μM) of UCB-J and UCB2400 were then prepared by diluting the stock solution in H_2_O and applied as 1 μL spots onto naïve sagittal rat brain sections. Following a 1-h incubation in the desiccator, the rat brain sections were washed with a solvent of choice within a range of certain polarity indexes (PI). The solvents used were EtOAc (PI = 4.4), acetone (PI = 5.1), DCM (PI = 3.1) and 70% ethanol (PI = 6.3).^23^ The tissue sections were washed by immersing the slides in solvent for 30 s. This duration is also consistent with previous studies on lipid removal, which recommend washing times between 15 and 30 s.^21,24,25^ Slides were placed vertically in staining beakers and solvent was added until the slides were fully immersed, utilizing approximately 65 mL of washing solvent for this purpose. The glass slide was subsequently placed in a desiccator until completely dry (10 min), as the use of organic solvents allows for rapid drying. The DHB matrix was then sublimed onto the tissues spiked with UCB-J and UCB2400 using the parameters listed in Table 1. The experiments were performed in triplicate.

#### Tissue Homogenate Matrix Application

For imaging of tissue homogenates (biological replicates n = 3), sublimation of DHB was performed at 160 °C for 160 s for both UCB-J and UCB2400. Tissue homogenates were immersed in DCMor EtOAc for 30 s as a washing step, and after washing, the samples were dried in a desiccator until completely dry before matrix application. Experiments were performed in triplicate for each biological replicate.

#### Comparison of Washing Solvents

One dosed brain tissue section was immersed in either DCM or EtOAc as described previously. Following immersion, a SpeedVac was employed to completely dry out the solvent. The dried residue was then resuspended in 1 mL of either DCM or EtOAc and spotted onto a glass slide. Subsequent to this, DHB was sublimated onto the sample as described in Table 1.

### Mass Spectrometry Imaging

All measurements were performed using a tims-TOF fleX instrument (Bruker Daltonics Inc.) at a raster size of 50 × 50 μm. The instrument was operated in positive mode in the mass range of *m*/*z* 250–500 with 100 laser shots per pixel at a frequency of 1000 Hz and optimized laser fluency.

### Data Analysis

Data processing was performed in SCiLS lab software (SCiLS, Bremen, Germany). Root mean square (RMS) normalization was employed to calculate the intensity values of UCB compounds for matrix selection where we calculated the μg UCB compound/g brain based on the size of the spot and the concentrations spotted. For further data analysis, normalization to a matrix peak (DHB, [2M+H-2H2O]^+^*m*/*z* 273.04) was used. The intensity was set to the average, and the interval processing mode was set to the peak maximum. Average peak intensities were exported from SCiLS and divided by the area of the homogenates or regions in the brain to create calibration curves and calculate the concentrations. Ion images of the compounds were obtained using SCiLS. LOD was calculated based on signal-to-noise ratio >3.^26,27^

### Tissue Staining

Rat brain sections were stained post MALDI-MSI with hematoxylin and eosin (H&E). A step-by-step description of the staining process is provided in Supporting Information.

### Immunohistochemistry (IHC) Staining

For IHC experiments, sections were blocked for 30 min at room temperature (RT) in blocking buffer containing donkey serum (1:500) in TBS-T (TBS with 0.05% Triton X-100, Sigma-Aldrich, Zwijndrecht, The Netherlands). Sections were then incubated overnight at 4 °C with mouse anti-SV2 (1.55 μg/mL, DSHB #AB_2315387, 1:2000) in TBS-T.^28,29^ Next, sections were washed twice with TBS-T for 5 min and once with TBS for 5 min. Sections were then incubated with Alexa Fluor 594 donkey antimouse (1/300, 2 mg/mL, A21203, LOT 2474956, Invitrogen) and DAPI (1:100, CAS no 28718903, ROTH) in TBS-T for 1 h at RT. After incubation with the secondary antibody, the sections were washed three times as described above. The sections were mounted in Vectashield (H-1000, LOT ZK1204). Two glass slides were used and each contained four brain sections: two from the 5 min postdose time point and two from the 20 min postdose time point. In total, two biological replicates were tested for each time-point. For each time point, one section was treated with both primary and secondary antibodies, while the other was treated with only the secondary antibody to serve as a negative control. Whole-brain images were acquired using an Olympus BX50 microscope at 10× magnification, with exposure times of 1000 ms for the 594 nm and 10 ms for the 350 nm channels. The images were acquired and stitched using Stereo Investigator software (MBF biosciences).

Higher magnification images of the cortex and hippocampus were captured using an Olympus BX51 microscope at 20× magnification with identical exposure settings.

### PET Reference Study for Comparison

To assess the calculated concentrations in the dosed animals after MSI experiments, we performed a comparison using data from an internal UCB *in vivo* PET experiment on rats dosed intravenously at 0.1 mg/kg followed by LC-MS analysis. Concentrations observed at 5 min, 10 and 20 min postdose in this reference LC-MS study, were compared to the concentrations detected by MSI at 3 mg/kg. (Supporting Information Table S1)

Quantification of tissue regions was conducted using H&E staining in conjunction with MSI results. H&E-stained sections were overlaid to delineate specific areas of interest, facilitating the segmentation of distinct tissue compartments. For each defined region, three smaller regions were selected within and the average peak maximum intensity (after normalization to matrix peak DHB, [2M+H-2H_2_O]^+^*m*/*z* 273.04) of UCB-J for each smaller region was extracted from Scils. (Figure S2) This intensity was averaged per region and normalized to the area of the corresponding tissue region (measured in mm^2^). This methodology was applied across all biological replicates. The average and standard deviation of the UCB-J signal intensities were subsequently calculated to evaluate variability and reproducibility of the data.

## Results and Discussion

### Influence of Matrix Selection

The selection of an appropriate matrix is a critical step in maximizing ion signal and achieving optimal performance in MALDI-MSI.^30^ In this study, we assessed the influence of matrix choice on the detection and analysis of two PET tracers, UCB-J and UCB2400, with a particular emphasis on mitigating ion suppression effects attributed to the presence of other small molecules, such as lipids and metabolites.

Concentrations ranging from 50 μM to 1 μM of both PET tracers were manually spotted onto naïve rat brain sections, followed by matrix sublimation to evaluate the matrix performance. Our matrix selection encompassed three candidates: DHB, CHCA and DCTB, chosen based on their established efficacy in small molecule analysis and reported enhancements in sensitivity for specific compound classes. DHB and CHCA specifically were included due to their success in small molecule MALDI-MSI studies.^11,17,31−33^ The inclusion of DCTB stemmed from its ability to increase ion signal, particularly for compounds targeted at the central nervous system, suggesting its potential use in enhancing sensitivity in our experiments.^34^

Based on prior research demonstrating the use of solvent- and matrix-free methods for visualizing PET tracers, we selected sublimation as the matrix application method.^13,14^ Like solvent-free approaches, sublimation can be considered a drier method than commonly used techniques such as spraying. Further, preliminary studies on a SolariX FTC-ICR showed sublimation to yield better sensitivity compared to spraying (data not shown).

Our findings revealed distinct performance among the evaluated matrices. Both DCTB and DHB exhibited superior signal intensities for both PET tracers in positive mode, with DCTB demonstrating the highest signal intensity overall, as shown in Figures S3 and S4. The calculated LOD’s were 0.6 ± 0.22 μg UCB-J/g brain for CHCA, 0.78 ± 0.10 μg UCB-J/g brain for DHB and 0.05 ± 0.02 μg UCB-J/g brain tissue for DCTB, while for UCB2400 they were 3.70 ± 2.58 μg UCB2400/g brain for CHCA, 2.56 ± 0.73 μg UCB2400/g tissue for DHB and 0.39 ± 0.04 μg UCB2400/g tissue for DCTB. The calculated limit of quantification (LOQ), which was derived from the LOD, was 1.98 ± 0.73 μg UCB-J/g brain for CHCA, 2.57 ± 0.33 μg UCB-J/g brain for DHB, and 0.17 ± 0.07 μg UCB-J/g brain tissue for DCTB; for UCB2400, the LOQs were 12.21 ± 8.51 μg UCB2400/g brain for CHCA, 8.45 ± 2.41 μg UCB2400/g tissue for DHB, and 1.28 ± 0.13 μg UCB2400/g tissue for DCTB. However, DCTB resulted in heterogeneous and larger crystal sizes, which are not favorable for imaging (Figures S5 and S6). This underscores the need for further refinement and optimization of sublimation parameters to investigate the full potential of DCTB in enhancing the signal intensity and spatial resolution in future MALDI-MSI studies. Although better sensitivity was achieved using DCTB, we proceeded with DHB as our matrix of choice for both PET tracers.

### Organic Washing Solvents Influence the Signal of the Compounds

The above-mentioned experiments led us to identify the most suitable matrix for PET tracer detection. However, the presence of numerous endogenous biomolecules in tissues poses a challenge as they could suppress the signal of PET tracers at clinically relevant concentrations. Ion suppression is a major challenge in MSI for drug imaging arising from various molecules due to the broad detection capability of MSI.^35^ These factors include the physical properties and ionization processes of the sample on PET tracer extraction, desorption and ionization.^35,36^ To address this issue, washing procedures can be employed to reduce the suppression impact and enhance the sensitivity for detecting PET tracers.^17,17^

Washing procedures using organic solvents were explored to selectively remove unwanted molecules while preserving the target compounds (UCB-J and UCB2400). We selected different washing solvents, respectively EtOAc, DCM, acetone and 70% ethanol, based on previous studies showing their effectiveness in enhancing the signal of metabolites and proteins by removing lipids.^20,25,37−39^ Both UCB compounds can cross the blood-brain barrier (BBB), indicating a lower degree of polarity.^40,41^ Given their high binding affinity to their targets as they cross the BBB, we hypothesized that applying an organic solvent washing step would selectively eliminate lipids while preserving the tracer binding. To ensure the preservation of the specific binding, solvents were carefully chosen based on varying polarities.^42^

**Figure 1:**
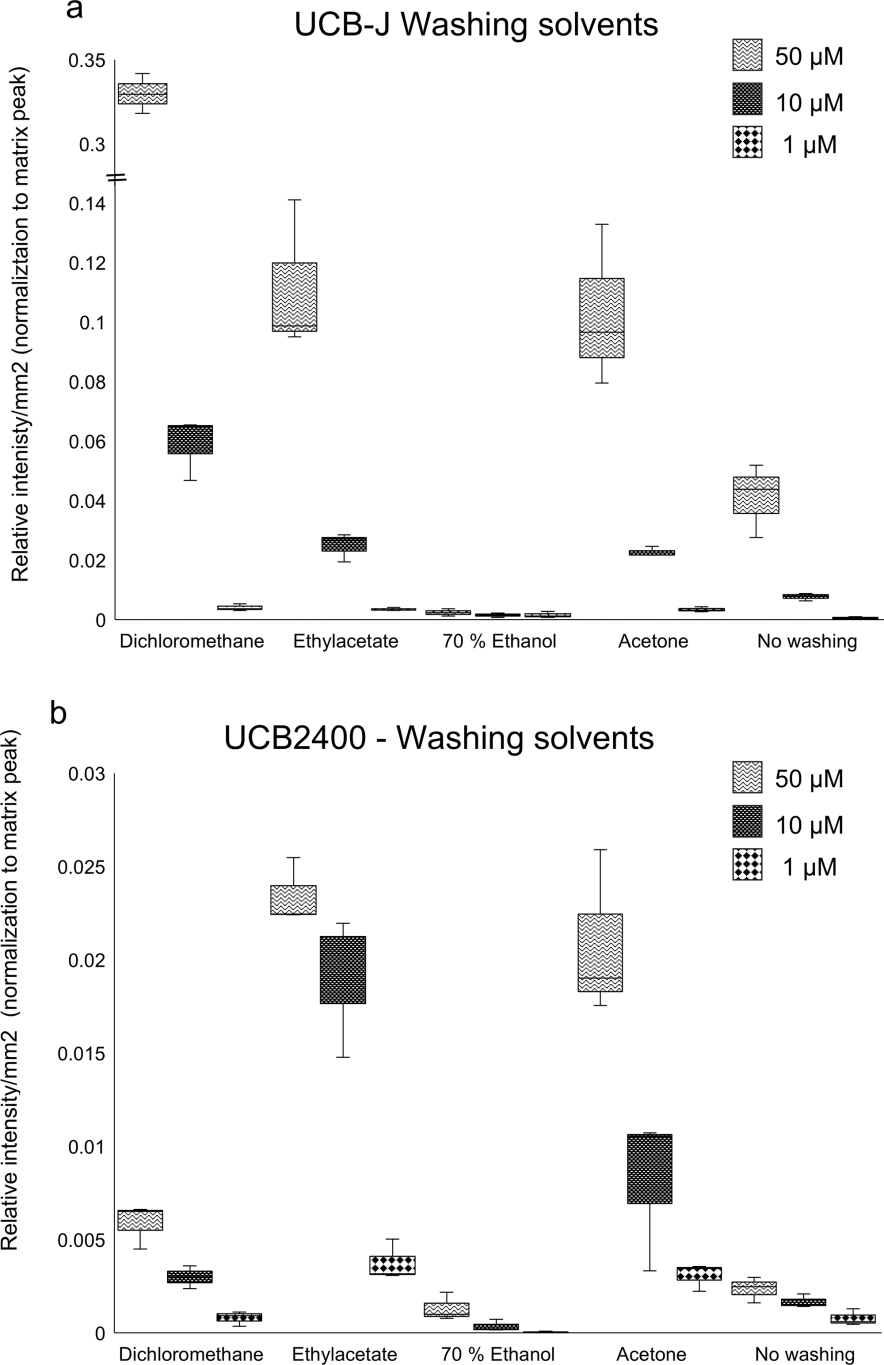
Three different concentrations (50, 10, and 1 μM) were spotted on naïve rat brain tissue for (a) UCB-J and (b) UCB2400, followed by sublimation of DHB. Different washing steps were tested including DCM, acetone, EtOAc, and 70% ethanol. Relative intensity/mm^2^ of the spots is normalized to the matrix peak (DHB, [2M + H– 2H_2_O]^+^*m*/*z* 273.04).

Initial experiments involved manually spotting of both UCB compounds onto naïve rat brain tissue followed by incubation and washing with various organic solvents, which is an straightforward way to gain a preliminary understanding of the signal on tissue and ensure detection of the compound. Figure 1 illustrates the signal intensities of three different concentrations of each UCB compound after washing. The results, depicted in the boxplots, demonstrate that DCM significantly enhanced the signal intensity for UCB-J, yielding a 10-fold increase compared to the no washing condition at 50 μM. In contrast, EtOAc exhibited higher signal intensities for both UCB-J and UCB2400 than acetone, indicating its overall effectiveness as a washing solvent. However, for UCB2400 at 10 μM, there is a notable deviation from linearity. This emphasizes the preliminary nature of these spotting experiments, which guided our next decision to utilize homogenates for a more accurate representation of the *in vivo* situation. Acetone yielded similar results as EtOAc, however due to acetone being more volatile, we continued with EtOAc, which allows for a more controlled drying process and ensures greater reproducibility and reliability in our results. Furthermore, while 70% EtOH was used as a washing agent, it resulted in negligible signals for both UCB-J and UCB2400, suggesting that it does not aid in suppression of the signal of other ions (lipids).

Although manual spotting provides insights into the potential efficacy of organic solvents as washing agents, caution is required. The less polar properties of DCM could also potentially wash less lipid compounds, resulting in more ion suppression of UCB compounds, which cannot be observed by manual spotting of higher concentrations on top of the tissue. The above-mentioned experiments provide thus a basis for detection of the PET tracers by MSI. However, they lack the ability to mimic the *ex vivo* conditions accurately ea. dosed animals.

### Homogenates Spiked with PET Tracers

**Figure 2:**
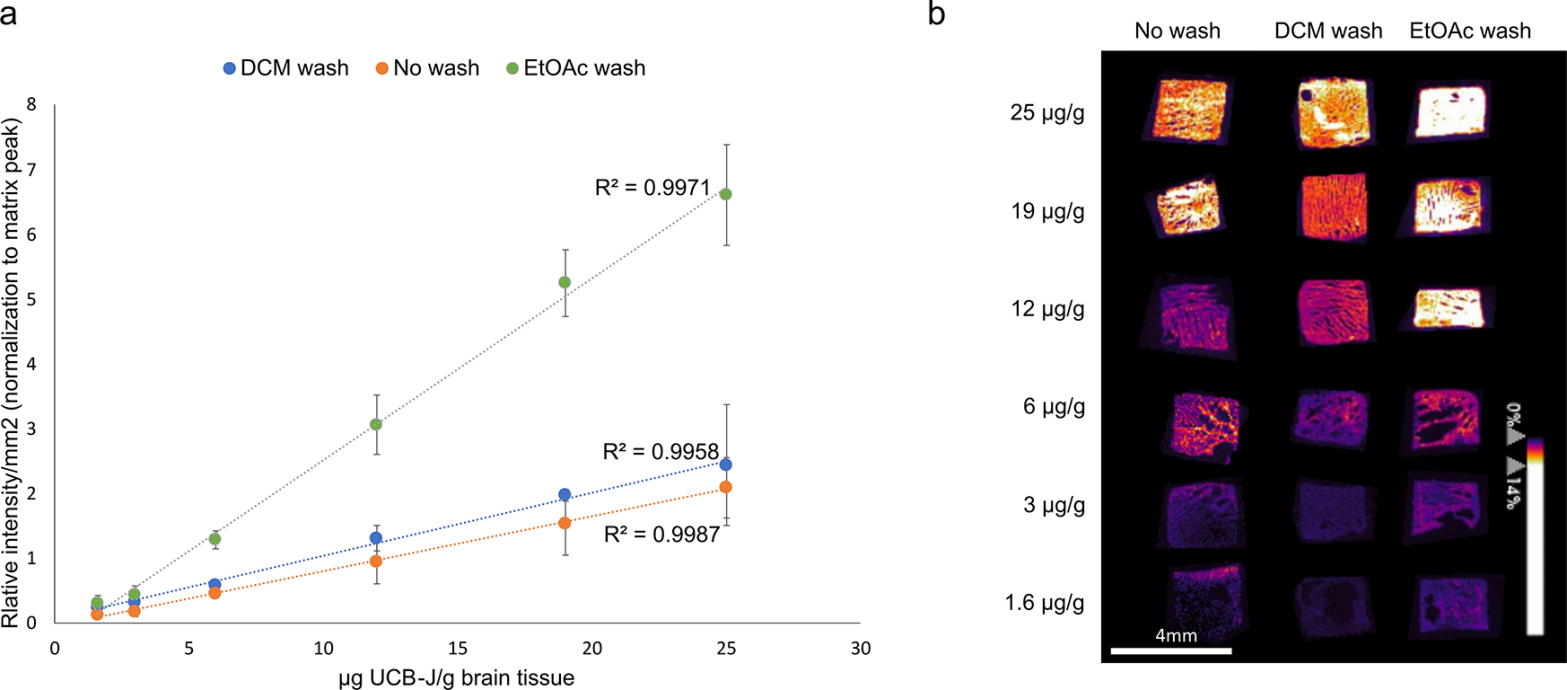
(a) Calibration curves plotting the signal of μg UCB-J/g brain tissue over the relative intensity/mm^2^ normalized to the matrix peak (DHB, [2M + H – 2H_2_O]^+^*m*/*z* 273.04) for each biological replicate. Homogenates were measured in triplicate. The error bars correspond to the standard error. (b) Representative MALDI-MSI images of the different homogenates spiked with the UCB-J (in μg UCB-J/g brain tissue) after no washing, a DCM wash, and anEtOAc wash.

To address the limitations of the manual spotting experiments, the next step involved preparation of brain homogenates spiked with known concentrations of both UCB compounds (UCB-J and UCB2400). To conduct these experiments, we collected naïve rat brains, homogenized them, and added known concentrations of either UCB-J or UCB2400. For both compounds, these concentrations ranged from 1.6 μg/g brain tissue to 25 μg/g brain tissue. In both cases, homogenates were measured after DCM washing and after EtOAc washing and were compared with unwashed homogenates. Figure 2 depicts the obtained calibration curves and the accompanying ion heat maps for UCB-J. The calibration curves showed clear linearity, with coefficients of correlation R^2^ > 0.995. Comparable results were obtained for UCB2400 (Figure S7). At concentrations below 5 μg UCB-compound/g brain tissue, deviations impacting the R^2^ values were observed. These deviations are likely due to variations in ionization efficiency at lower concentrations, rather than true nonlinearity. Such behavior has been reported in previous studies utilizing quantitative MSI,^43,44^ where lower ionization efficiency at low compound concentration levels can affect quantification accuracy. This reflects the inherent challenges in achieving consistent ionization for low-abundance compounds rather than indicating a genuine deviation from linearity.

Further, the standard deviations within these experiments can be primarily explained by the absence of an internal standard for normalization.^43,45−49^ In this particular context, the preparation of an internal standard that could serve as a reference point for calibration, was not feasible due to the associated costs. Consequently, normalization of the UCB-compound signal was performed with a matrix peak.^50^ Additionally, it is important to consider that the mimetics may exhibit variability due to freezing artifacts and air bubbles.^51^

Overall, the calibration curves clearly demonstrated that both the DCM and EtOAc washing procedures resulted in increased signal intensity. Specifically, the signal enhancement was more pronounced following an EtOAc wash, showing a 3-fold increase compared to the no washing samples, whereas DCM washing led to only a 1.4-fold increase. The EtOAc wash consistently displayed higher signal intensities, particularly at low concentrations, with an LOD of 0.34 ± 0.05 μg UCB-J/g brain tissue. In comparison, unwashed sections had a LOD of 1.63 ± 0.28 μg UCB-J/g brain and DCM wash had a LOD of 0.27 ± 0.03 μg UCB-J/g brain. The calculated LOQs, were 1.12 ± 0.17 μg UCB-J/g brain tissue for the EtOAc wash, 5.28 ± 0.92 μg UCB-J/g brain for unwashed sections, and 0.89 ± 0.10 μg UCB-J/g brain for DCM wash. For UCB2400, the calculated LODs were 0.01 ± 0.002 μg UCB-2400/g brain for EtOAc wash, 0.58 ± 0.47 μg UCB-2400/g brain for not washed and 2.34 ± 1.13 μg UCB-2400/g brain for DCM wash. The calculated LOQs were 0.033 ± 0.007 μg UCB-2400/g brain for the EtOAc wash, 1.91 ± 1.55 μg UCB-2400/g brain for the unwashed sections, and 7.72 ± 3.73 μg UCB-2400/g brain for the DCM wash. These calculations suggests that EtOAc is more efficient for analyte detection, especially at the selected concentrations, although the LOD of EtOAc for UCB-J is higher compared to DCM, which can be attributed to previous mentioned ionization efficiency at lower concentrations. In addition, the absence of signal with 70% ethanol in the manual spotted experiments further suggests the possibility that not enough lipids are removed, which further substantiates this hypothesis. To delve deeper into these findings, we conducted further analysis on dosed rat brain tissues of UCB-J.

### Distribution of PET Compounds in Rat Brain

#### UCB-J Distribution without Washing

**Figure 3:**
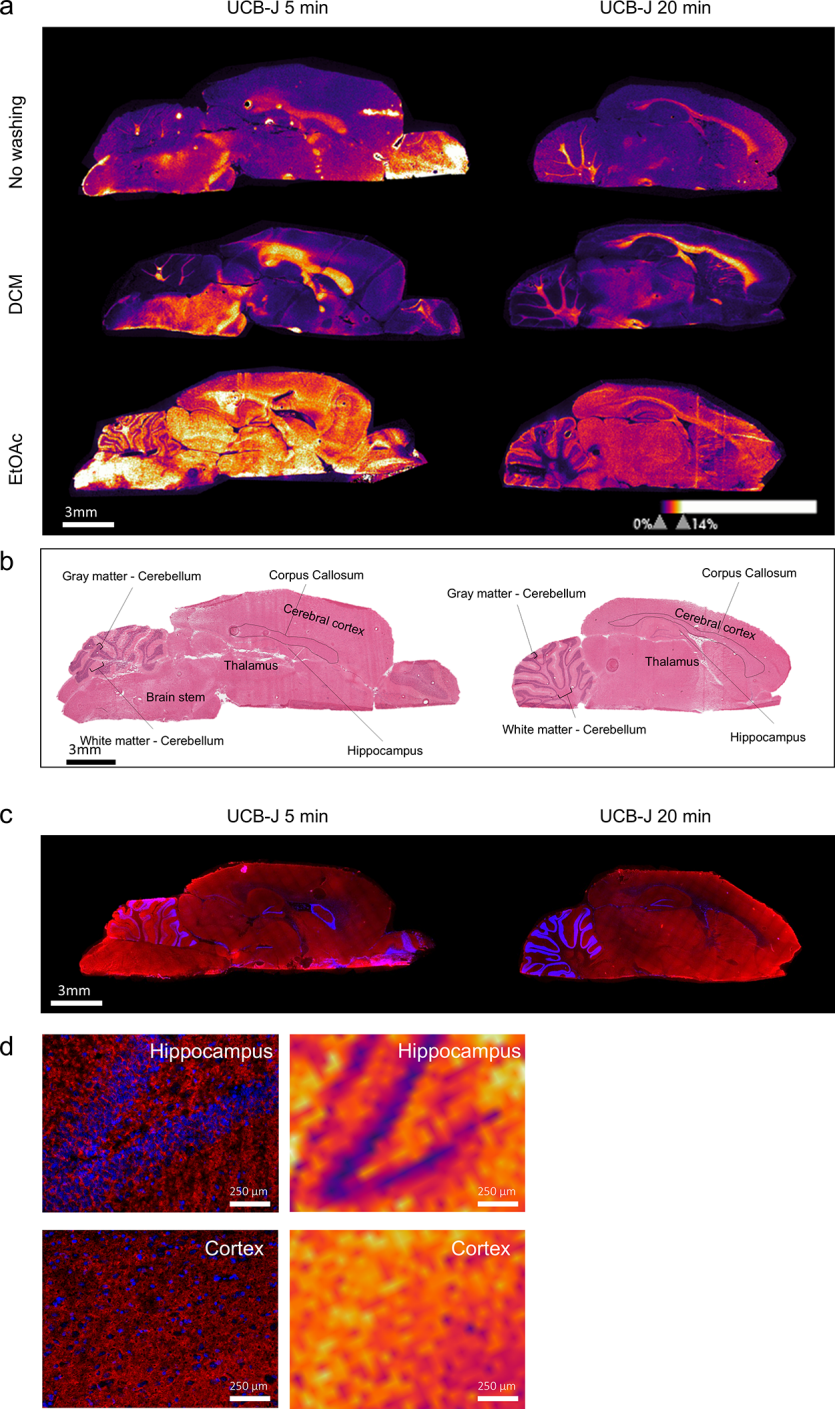
(a) UCB-J distribution in dosed animals. Distribution of UCB-J in animals 5 min and 20 min post-dose after no washing, a 30 s DCM washing step, or a 30 s EtOAc washing step together with (b) H&E staining of the corresponding rat brain tissue sections, with annotation of the different regions in the brain. (c) SV2A IHC staining (SV2A signal = red, DAPI = blue) showing the distribution of SV2A in consecutive brain sections. (d) Zoomed-in SV2A distribution of the hippocampus and cortex (left) next to zoomed-in MALDI-MSI images (right) of the same region in the dosed UCB-J brain (5 min post dose) showing the overlap of the SV2A distribution with the MALDI-MSI signal of UCB-J.

MALDI-MSI was conducted on rats administered with UCB-J at two postdosing time-points (5 and 20 min). In the absence of an organic washing solvent, UCB-J signals were detected in certain regions of the brain, with notably higher signal intensities observed in the corpus callosum and white matter of the cerebellum (Figure 3, no washing). Given that synaptic vesicle glycoprotein 2A (SV2A) has a very low abundance in these regions, as indicated by IHC staining in Figure 3c, the observed signals likely represent unbound tracer.

#### Effect of DCM and EtOAc Washing

Following a DCM washing step, UCB-J signals in these corpus callosum and white matter of the cerebellum exhibited a remarkable 2.5-fold increase in signal compared to no washing. Further, signal detection in the other brain regions was low to non after a DCM washing step. This similar distribution to no washing also indicated that the observed UCB-J signal is likely indicative of unbound tracer rather than specific binding to its target. These results further align with a prior study demonstrated that ion suppression by lipids is less pronounced in white matter compared to other brain areas, suggesting that a DCM washing step (or even no washing step) would result in signals primarily observed in white matter due to reduced ion suppression as observed in our data.^35^

**Figure 4:**
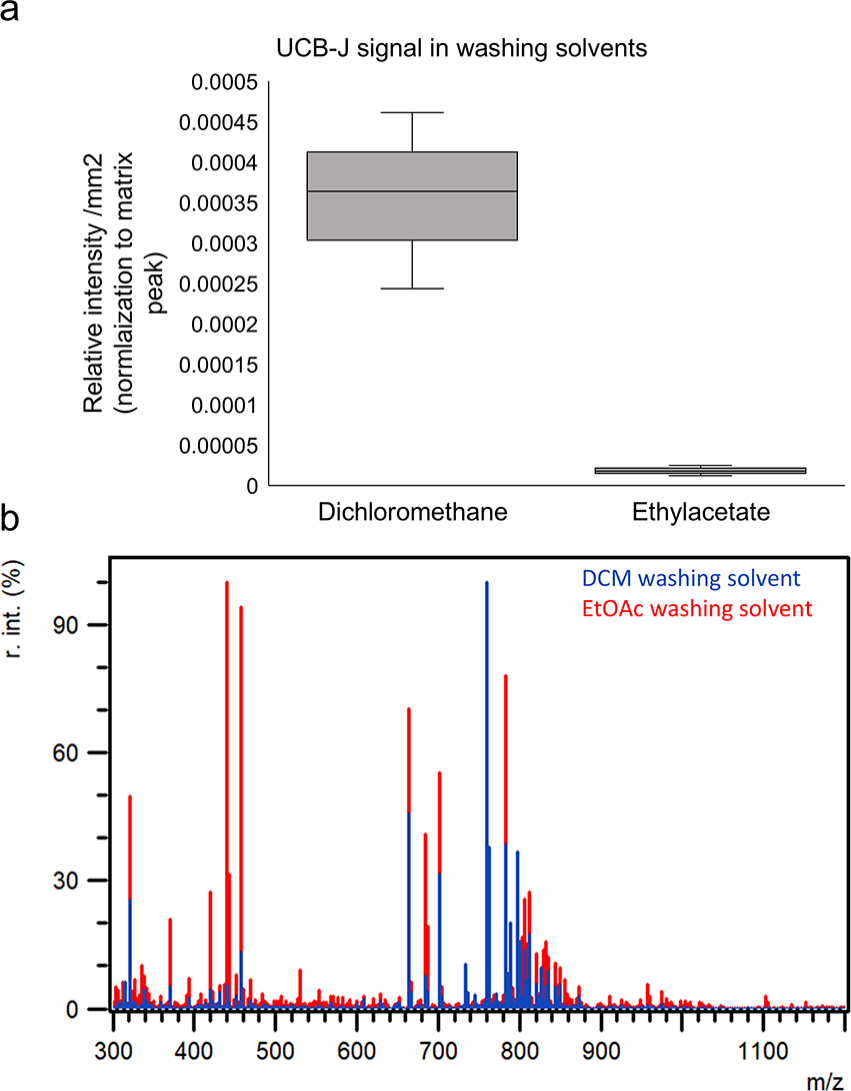
Signal of UCB-J and lipids in washing solvents. (a) Boxplot representing the signal (normalization to matrix peak (DHB, [2M + H – 2H_2_O]^+^*m*/*z* 273.04)) of UCB-J compound measured in spots of the washing solvents, including DCM and EtOAc. (b) Average spectra of the washing solvent after a DCM or a EtOAc washing step.

DCM is a solvent with a polarity index of 3.1, dissolving molecules such as less polar or neutral lipids, including di- and triglycerides, along with certain polar phospholipids.^52,53^ Hence, it serves as an ideal washing solvent for MALDI-MSI, where lipids can significantly contribute to ion suppression.^39^ Spectral analysis reveals abundant lipids in the washing solvent (specifically in the phospholipid range, 600–900 Da) and almost no to very little signal of UCB-J (Figure 4a and Figure S8). Further, DCM demonstrates the highest signal for LOD in both spotted tissue and spiked homogenates for UCB-J. We hypothesize that its robust ability to mitigate ion suppression, through the selective removal of lipids during washing, contributes to the elevated LOD observed^52^.

After applying an EtOAc washing step, the distribution pattern of UCB-J differed notably compared to both the no washing and DCM-washed conditions. Specifically, increased signal was observed in the gray matter of the cerebellum, thalamus and cerebral cortex regions of the brain. (Figure 3 and Figure S9).

EtOAc with a polarity index of 4.4, facilitates the removal of a broader spectrum of lipids, encompassing neutral lipids, lysolipids (400–600 Da), and polar phospholipids^53^ (Figure 4). This differential removal is evident in the mass spectra presented in Figure 4, which displays pronounced signals in the lysophospholipid range following EtOAc washing. The removal of lysophospholipids is particularly of interest because their masses are closer to that of UCB-J, reducing potential ion suppression effects in this mass range and improving ionization efficiency. This alignment between the mass of lysophospholipids and UCB-J suggests that their selective removal minimizes competition for ionization, thereby enhancing signal detection and overall sensitivity in the analysis.

Signal intensities of UCB-J in EtOAc washing solvent were lower than in the DCM washing solvent suggesting EtOAc appears to function as a balanced washing step, primarily eliminating more small molecules and lipids without removing the compound (Figure 4a). This balance is similar to autoradiography, where polar solvents like TRIS buffer or PBS are typically utilized to wash away unbound tracers.^54,55^

#### Comparison with IHC and PET Imaging

After conducting the EtOAc wash, we observed that the distribution of UCB-J closely matches the theoretical distribution of SV2A target protein, as indicated by an IHC staining performed by previous studies and our IHC staining showing the distribution of SV2A^56−59^ (Figure 3c,d and Figure S10). Our IHC staining shows that SV2A is extensively present in the gray matter of the brain in regions such as the cortex and hippocampus, with reduced signal in the white matter. (Figure 3d) Moreover, a previous report on UCB-J distribution in rats, using PET imaging, demonstrated similarities between the PET imaging results and the distribution observed via MALDI-MSI following an EtOAc wash step.^55,60^ Given these similarities, EtOAc emerges as the preferred choice for detection of the UCB-J PET tracer.

#### Validation of UCB-J Detection

Confirmation of UCB-J was achieved through targeted MS/MS, comparing the UCB-J standard solution with dosed rat brains after DCM and EtOAc washing step (Figure S11). Further confirmation was obtained by the lack of signal in control rat brains after no washing, DCM and EtOAc washing (not dosed) (Figures S12 and S13).

#### Quantification of UCB-J

In our final analysis, we quantified the concentration of UCB-J in specific brain regions using the optimized EtOAc washing protocol with the calibration curves of the above-measured homogenates. Our findings revealed that on average the concentration of UCB-J across the entire brain section was 12.11 ± 3.15 μg UCB-J/g brain tissue at 5 min postdose, decreasing to 5.42 ± 1.33 μg UCB-J/g brain tissue at 20 min postdose. Comparison with a reference study estimating UCB-J concentrations based on a low dose *in vivo* PET study yielded values of 11.09 μg UCB-J/g brain tissue at 5 min postdose and 7.5 μg UCB-J/g brain tissue at 20 min postdose, indicating a close similarity between our quantification results and the reference study (Supplementary Table S1). In the 20 min postdose comparison, a discrepancy is noted between our study and the reference study. Moreover, detailed analysis of specific brain regions in these sections showed concentrations of 9.90 ± 1.81 μg UCB-J/g brain tissue at 5 min postdose and 6.02 ± 2.22 μg UCB-J/g brain tissue at 20 min postdose in the gray matter of the cerebellum based on the calibration curves of the homogenates. For the white matter of the cerebellum, a concentration of 1.13 ± 0.05 μg UCB-J/g brain tissue 5 min postdose and 0.77 ± 0.17 μg UCB-J/g brain tissue 20 min postdose were calculated. Concentrations in the cerebral cortex were determined to be 11.08 ± 5.66 μg UCB-J/g brain tissue and 4.37 ± 0.69 μg UCB-J/g brain tissue for 5 and 20 min postdose, respectively. However, some limitations need to be considered with these quantifications. It is important to keep in mind that the reference values are extrapolated. Further, some calculated concentrations (e.g., in the white matter of the cerebellum after 5 and 20 min) fall below the LOQ, indicating that these levels may not be reliably detected using the current methodology.

These findings have several important implications, which is evident when comparing our observed distribution patterns with those in Thomson et al.^55^ Specifically, regions such as the corpus callosum and other white matter areas, which show minimal tracer signals by Thomson et al, exhibit higher tracer abundances in our results (Figure 3).^55^ One potential explanation is the increase in tracer dose used in this study. The higher dose likely led to saturation of the target, thereby modifying the distribution pattern within the brain. This elevated dose, relative to clinical PET studies, might also contribute to an increased clearance rate, potentially accounting for the observed differences. Additionally, this higher dose administered may have caused retention of the nonbound fraction of UCB-J, leading to signals in areas not highlighted by PET imaging or IHC staining of SV2A. The high dose of the tracer might further have influenced UCB-J’s ability to penetrate the BBB. At such a high dose, it is plausible that the tracer could exceed the capacity of BBB transport mechanisms or binding sites, potentially allowing greater penetration into the brain than would be observed at standard doses. The choice of washing solvent, EtOAc, further influences these observations. While EtOAc effectively removes a significant amount of lipids–thereby reducing lipid-related ion suppression, it does not allow for precise quantification of the nonbound and bound tracer. As a result, tracer signals are observed in areas such as the corpus callosum and brain stem where very little SV2A presence is expected as seen in the IHC staining (Figure 3).

However, these observations also support the hypothesis that the use of EtOAc as a washing solvent effectively removes the lipids without removing the compound, resulting in the detection of the UCB-J compound in the brain. A follow up experiment could be conceived using a PET tracer such as UCB2400, which has a very specific distribution in the brain, rather than a more ubiquitous presence like UCB-J to further investigate this phenomenon. After washing, this study could focus on measuring either free or bound fractions specifically in the restricted areas where UCB2400 is expected to localize.

Overall, the presented results have demonstrated the critical role of sample preparation in MALDI-MSI experiments, particularly in the context of PET tracer studies. A comprehensive workflow has been outlined, encompassing matrix selection and the crucial step of washing. Our finding further suggests the potential efficacy of an EtOAc washing step, as demonstrated in both manual spotted samples and homogenates of two different PET tracers, UCB-J and UCB2400. This underscores the capability of MALDI-MSI in PET tracer detection. Nevertheless, challenges such as ion suppression persist in MALDI-MSI, albeit mitigated to some extent through optimized washing steps, resulting in overall signal enhancement. It is imperative to note that while EtOAc has proven effective for the UBC-J tracer dosed animals, its applicability to other PET tracers remains unexplored. Therefore, future investigations should prioritize assessing the suitability of an EtOAc washing step for the detection of PET tracers not described in this paper in dosed animals.

Further, the differential effects of these washing solvents across various brain regions require further studies given the heterogeneous nature of brain tissue. Careful consideration must be given to the balance between lipid removal and compound retention. In addition to lipids, salts could also interfere with ionization and disrupt signal intensity. Despite the focus on lipids in the evaluation of ion suppression, other factors, such as salt ions might also contribute to signal disruption and should be considered in future studies to provide a more comprehensive understanding. If water-soluble compounds are of interest, caution is advised during desalting procedures, as analytes may be lost in the process. Lipids can generally be removed without affecting these compounds, as demonstrated by the solvents used in this study. Despite all limitations, the technique offers nonradioactive alternatives, that aid in decision making in drug development studies by the quantitative visualization of tracer distributions.

## Conclusions

This study highlights the crucial role of sample preparation in maximizing the efficacy of MALDI-MSI experiments, particularly in the context of brain PET tracer studies. Through matrix selection and washing procedures, we aimed to reduce ion suppression effects and enhance the detection sensitivity of two brain PET tracers UCB-J and UCB2400. Our findings underscore the significance of solvent choice in washing steps, with EtOAc emerging as a promising agent for effectively removing unwanted molecules while preserving target compounds in dosed animal models. Despite challenges such as ion suppression, our study demonstrates the potential of MALDI-MSI as a valuable tool for PET tracer detection, offering nonradioactive alternatives and enabling visualization of PET tracer distribution in drug development studies, although at higher concentrations compared to autoradiography. This makes MALDI-MSI particularly useful for early stage drug screening, where higher compound concentrations are typically required, with the option to transition to more sensitive methods for clinical applications at lower doses.

## Supporting Information Available

Chemical structures of UCB compounds, regions for quantification, UCB-J matrix selection, UCB2400 matrix selection, matrix crystals, UCB-J signal after DCTB sublimation, UCB2400 homogenates, UCB-J mass range spectrum of washing solvents, biological replicate-dosed animals, SV2A staining of control and dosed rat brains, MS/MS UCB-J compound, UCB-J mass range spectrum of control animals, signal of UCB-J in control animals, H&E staining protocol, and reference study calculations (PDF)
