# Supplementary material for: Spatial Distribution of Brain PET Tracers by MALDI Imaging
Source: J Am Soc Mass Spectrom. 2025 Mar 12;36(4):688–98. doi: 10.1021/jasms.4c00307 (PMC11969657; doi:10.1021/jasms.4c00307)
Supplement: Supplementary file 1 — js4c00307_si_001.pdf [file js4c00307_si_001.pdf]

Supporting Information

Spatial distribution of brain PET tracers by MALDI imaging

Isabeau Vermeulen[$], Michiel Vandenbosch[$], Delphine Viot[&], Joel Mercier[§], Diego Asensio-Wandosell Cabañas [%], Pilar Martinez-Martinez[%], Patrick Barton[»], Ron M.A. Heeren[$,#] and Berta Cillero-Pastor[$,+,#,*]

[$]The Maastricht MultiModal Molecular Imaging Institute (M4i), Division of Imaging Mass Spectrometry, Maastricht University, Universiteitssingel 50, 6229 ER Maastricht, The Netherlands
[&]Translational Science, DMPK, UCB Biopharma SRL, Chemin du Foriest, B1420, Braine-l'Alleud, Belgium
[§]Discovery Chemistry BE, UCB Biopharma SRL, Chemin du Foriest, B1420, Braine-l'Alleud, Belgium
[%]Department of Psychiatry and Neuropsychology, Universiteitssingel 40, 6229 ER, Maastricht University, Maastricht, the Netherlands.
[»]Translational Science, DMPK, UCB Celltech, Branch of UCB Pharma S.A., 208 Bath Road, Slough, Berkshire, SL1 3WE, United Kingdom
[+]Cell Biology-Inspired Tissue Engineering (cBITE), MERLN, Maastricht University, Universiteitssingel 40, 6229 ER Maastricht, Netherlands

Corresponding author: b.cilleropastor@maastrichtuniversity.nl

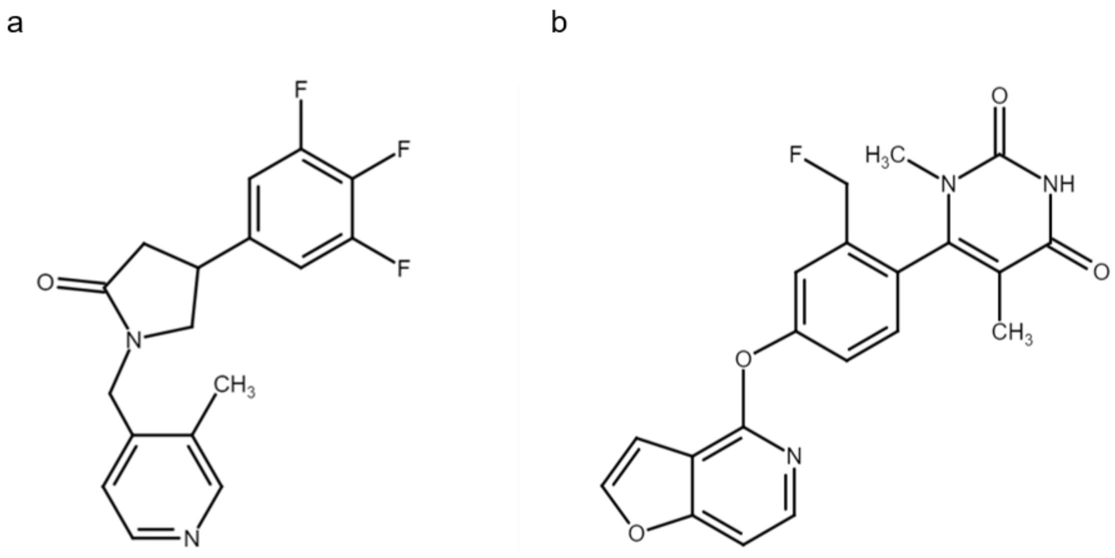

Supplementary figure S1. Chemical structure of UCB-J (a) and UCB2400 (b).

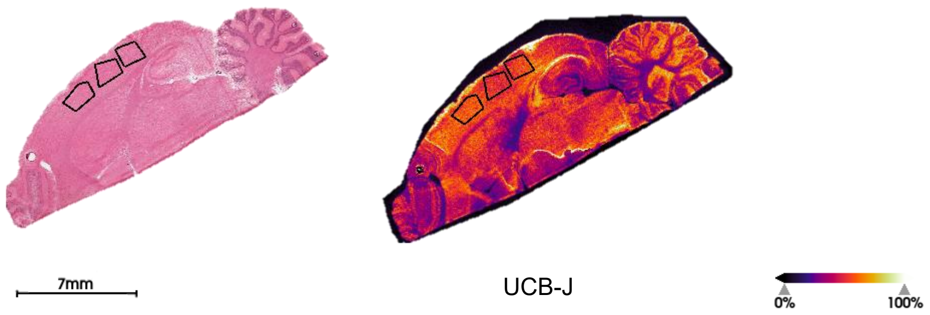

Supplementary Figure S2. Regions for quantification were selected based on haematoxylin and eosin staining (left), where three representative areas were identified within the cortex. These regions were then overlayed with the MALDI-MSI data for quantification, with the average signal from these areas used to approximate the cortical distribution.

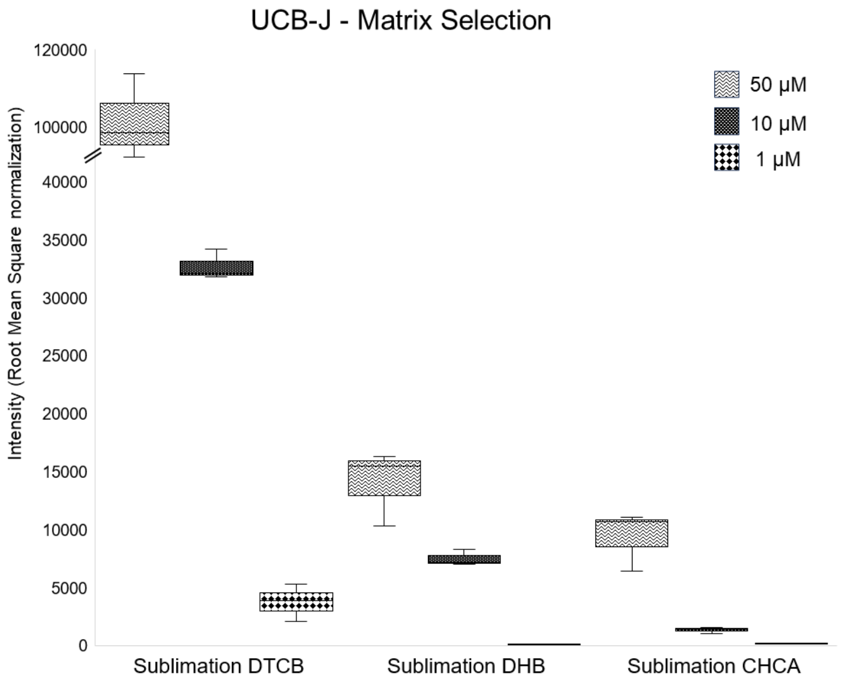

Supplementary figure S3. Signal of UCB-J spotted on naïve brain tissue after sublimation with CHCA, DHB or DCTB.

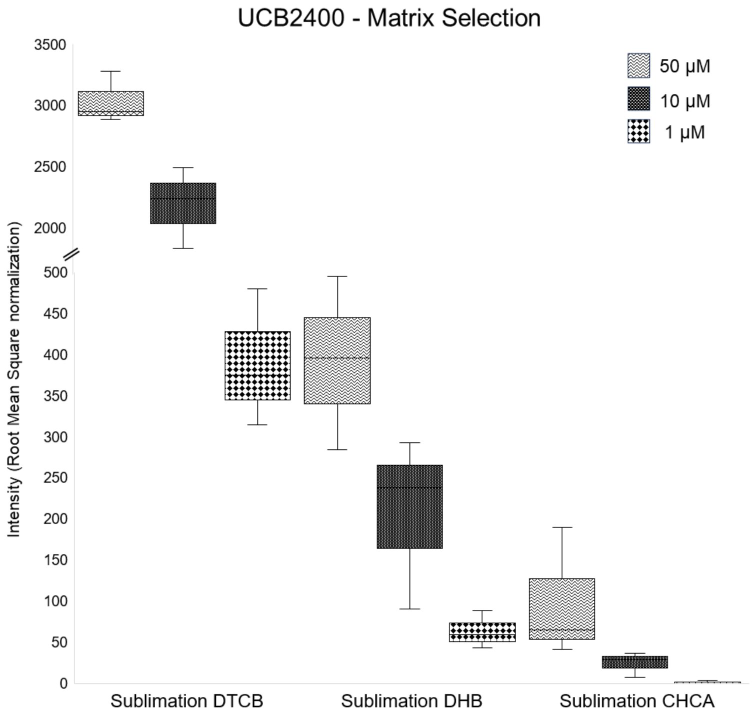

Supplementary figure S4. Signal of UCB2400 spotted on naïve rat brain tissue after sublimation with CHCA, DHB and DCTB.

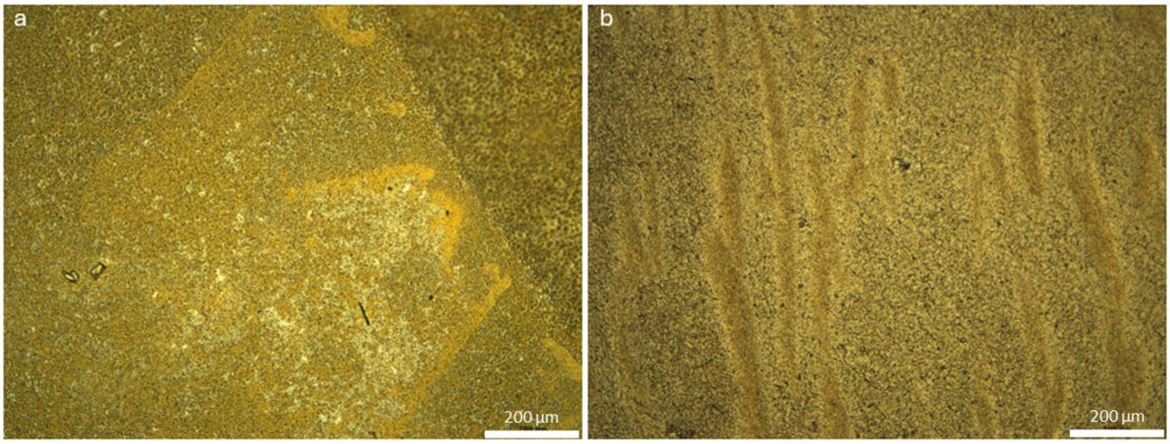

Supplementary figure S5. Microscope images (10X) of DCTB matrix on tissue (a), and DHB matrix on tissue (b) showing the larger crystal size in DCTB compared to DHB.

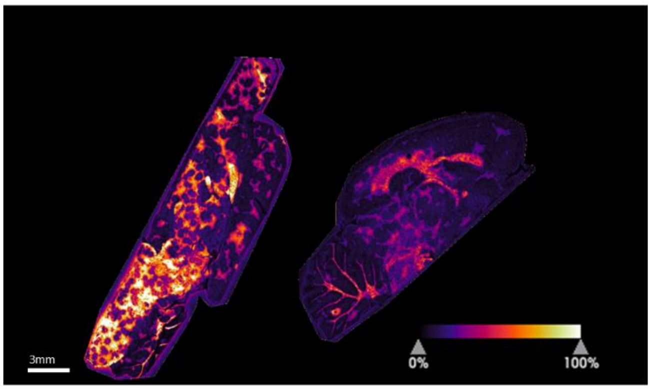

Supplementary figure S6. Signal of UCB-J in dosed rat brain (5 min post dose left and 20 min post dose right) after DCTB sublimation

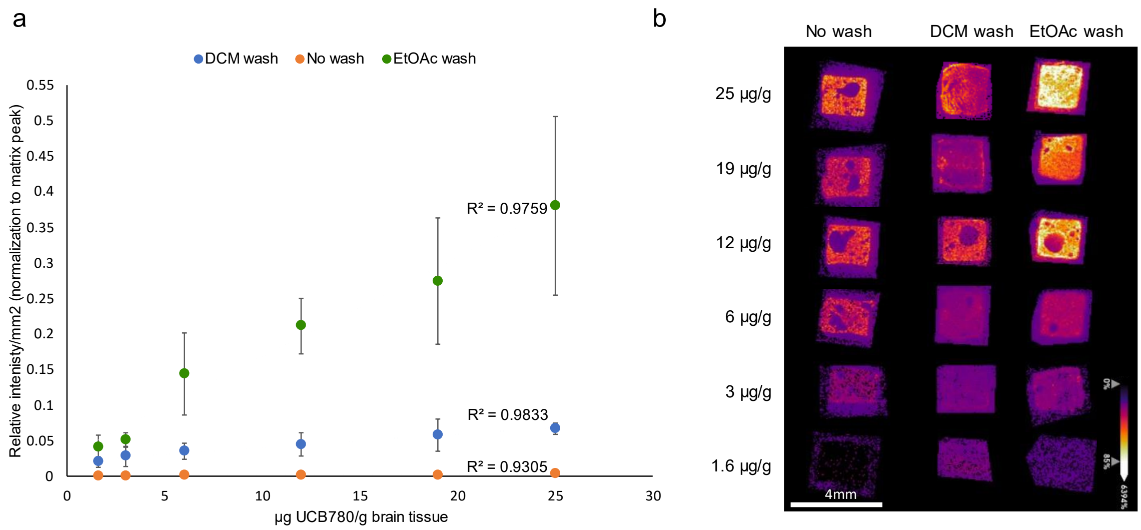

Supplementary figure S7 (a) Calibration curves plotting the signal of µg UCB2400/g brain tissue over the relative intensity/mm² normalized to the matrix peak (DHB, $[2M+H-2H2O]^+$ m/z 273.04) for each biological replicate. Homogenates were measured in triplicate. The error bars correspond to the standard error. (b) Representing MALDI-MSI images of the

different homogenates spiked with the UCB2400 (in µg UCB2400/g brain tissue) after no washing, dichloromethane (DCM), ethyl acetate (EtOAc).

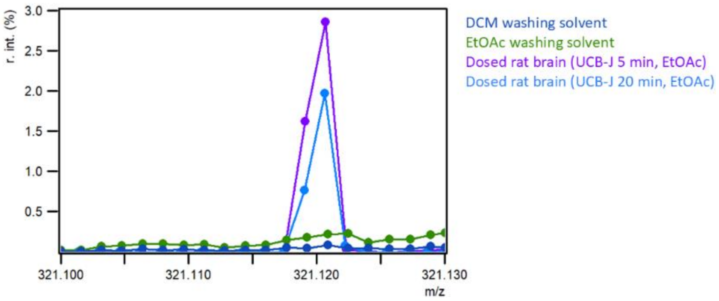

Supplementary figure S8: Average spectra (zoomed in on UCB-J mass range) of the dichloromethane (DCM) and ethyl acetate (EtOAc) washing solvents compared with dosed animals after EtOAc wash. The spectra clearly show a very low signal in both washing solvents.

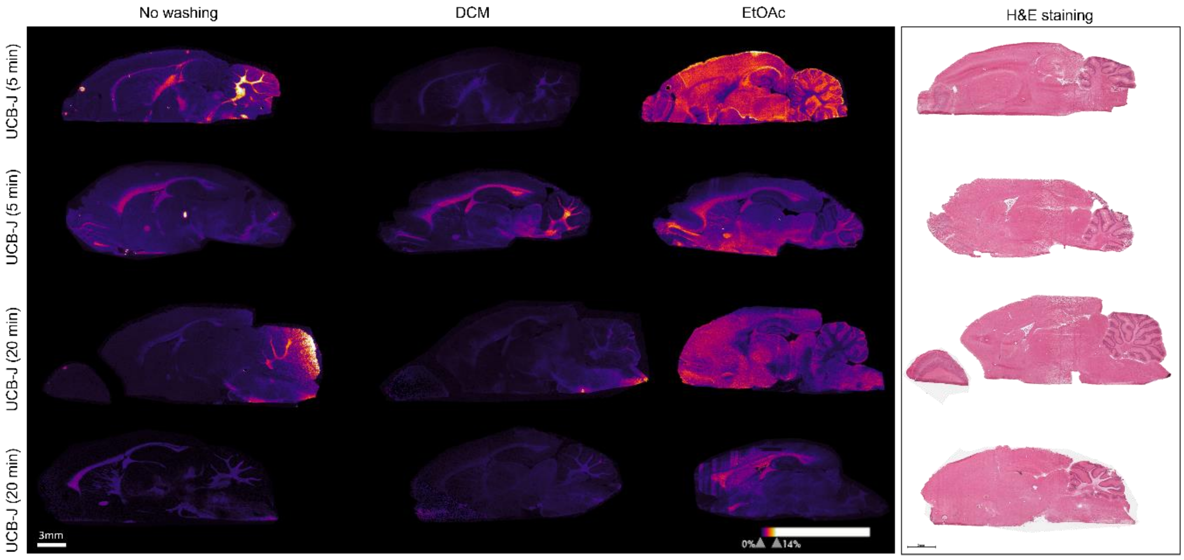

Supplementary figure S9. Biological replicates of dosed rat brain sections (5 min and 20 min post dose) without washing, after dichloromethane (DCM) wash and after ethylacetate (EtOAc) wash together with haematoxylin and eosin stainings.

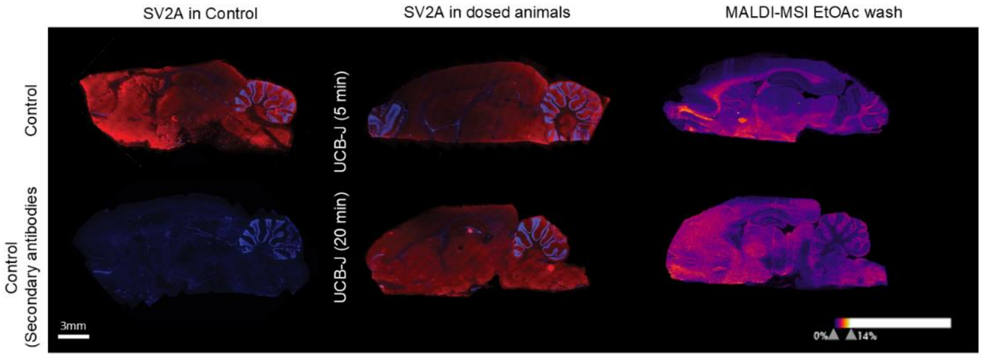

Supplementary figure S10. SV2A immunofluorescence stainings of control sections (including a control with only secondary antibodies and biological replicates of dosed rat brain sections (5 min and 20 min post dose) after a SV2A staining next to MALDI-MSI signal of UCB-J after an ethylacetate (EtOAc) washing step

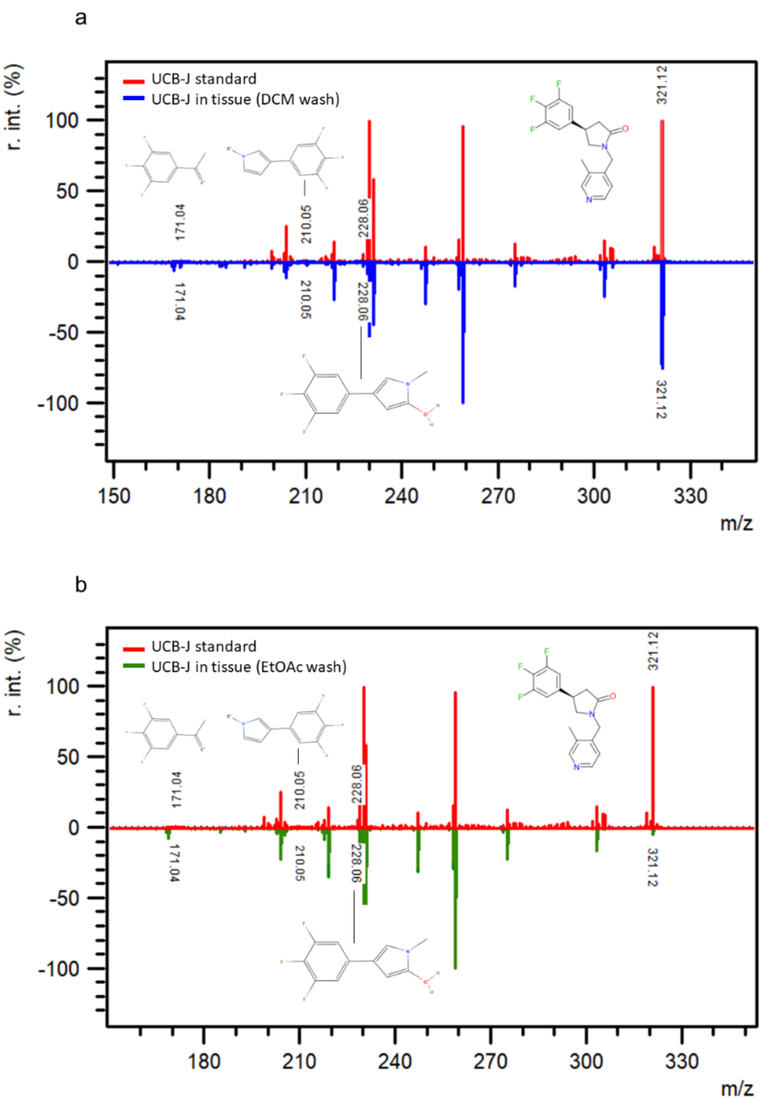

Supplementary figure S11. Confirmation of UCB-J by targeted MS/MS on both UCB-J standard solution and UCB-J in dosed rat brain tissue after a dichloromethane (DCM) wash (a) and UCB-J standard solution and UCB-J in dosed rat brain tissue after an ethylacetate (EtOAc) wash (b) showing multiple matching fragments of UCB-J.

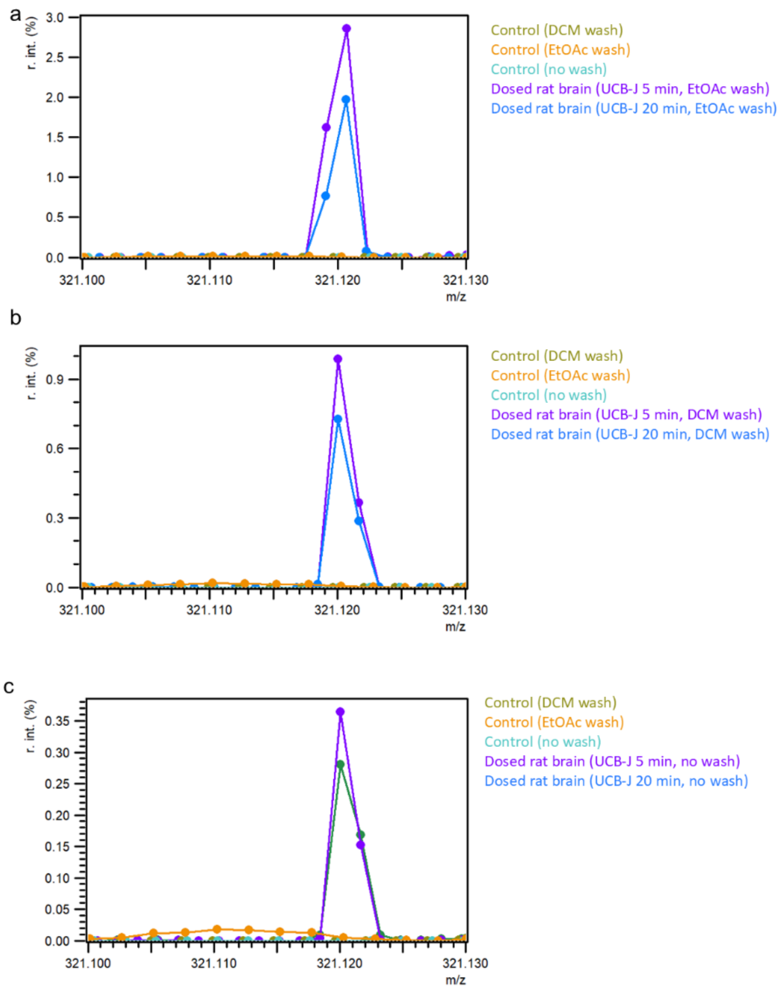

Supplementary figure S12: Average spectra (zoomed in on UCB-J mass range) of the Control animals after no wash , dichloromethane (DCM) wash and ethylacetate (EtOAc) wash compared with dosed animals after EtOAc wash (a), DCM wash (b) and no wash (c). The spectra clearly show a lack of signal in all control animals.

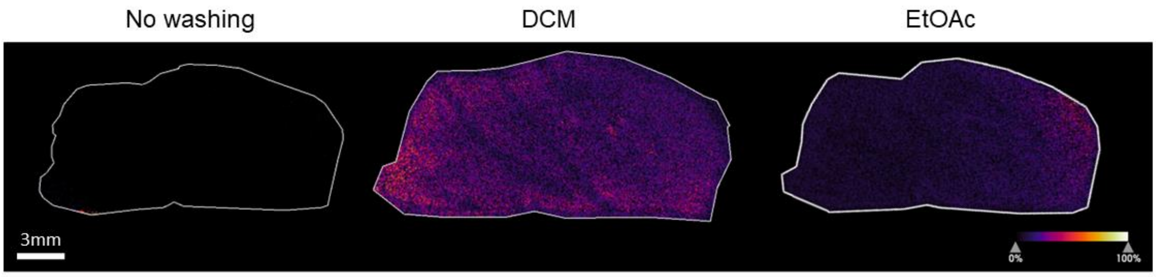

Supplementary figure S13. Control rat brain sections after no wash, dichloromethane (DCM) and ethyl acetate (EtOAc) washing showing little to no signal of UCB-J.

Hematoxylin-Eosin staining

Haematoxylin-Eosin staining's were performed on dosed animals. Slides were hydrated in distilled tap water for 3 min followed by haematoxylin (0.1% Gill's) staining for 3 min. Next, they were rinsed in running tap water for 3 min followed by a short rinse in distilled water. Slides were then stained with eosin (0.2%) for 30 sec followed by a short rinse in 70% ethanol to remove the excess of eosin. Next, the slides were dehydrated in 100% ethanol for 2 min (2x) and equilibrated in xylene for 5 minutes (2x). The stained sections were mounted with entellan and covered with a glass coverslip, followed by drying at RT. The stained sections were scanned with a digital scanner (Aperio CS2) at 20x magnification.

PET reference study

Based on a previous internal reference study from UCB-pharma, we estimated the expected concentrations at 3 mg/kg by extrapolating from the known concentrations at 0.1mg/kg. We then compared these expected concentration of UCB-J in the brain with our MALDI-MSI results. Initial concentrations were given in ng/mL. Given the density of brain is around 1g/mL, the concentrations are given in ng/g.

Table S1: Calculations reference study

**Design**	**Dose**	**Brain conc 5 min**	**Brain conc 10 min**	**Brain conc 20 min**
	mg/kg	ng/g	ng/g	ng/g
Ref study	0.1	410	225	265
	mg/kg	µg/g	µg/g	µg/g
Prediction	3.0	11.6	6.35	7.50
